# Application of wrapper based hybrid system for classification of risk tolerance in the Indian mining industry

**DOI:** 10.1038/s41598-023-32693-3

**Published:** 2023-04-15

**Authors:** Deepak Kumar, Ram Madhab Bhattacharjee

**Affiliations:** 1 Department of Mining Engineering, Indian Institute of Technology (Indian School of Mines), Dhanbad, 826004 India; 2 Bharat Coking Coal Limited, Dhanbad, 826004 India

**Keywords:** Energy science and technology, Engineering

## Abstract

The degree to which an individual is willing to take risks i.e., risk tolerance is often cited as a significant causal element in the majority of workplace accidents. It is essential to determine the risk tolerance level of miners and utilise their risk profiles to design improved training modules, safety, recruitment, and deployment policies. This paper aims to identify the most critical factors (or features) influencing miners’ risk tolerance in the Indian coal industry and develop a robust prediction model to learn their risk tolerance levels. To do end, we first conducted a questionnaire survey representing the complete feature set (with 36 features) among 360 miners and divided their responses into five classes of risk tolerance. Next, we propose a wrapper based hybrid system that combines particle swarm optimization (PSO) and random forest (RF) to train a multi-class classifier with a subset of features. In general, the proposed system selects the best feature subset by iteratively generating different feature combinations using the PSO and training an RF classifier model to assess the effectiveness of the generated feature subsets for the F1-score. At last, we compared the PSO-RF with four traditional classification methods to evaluate its effectiveness in terms of precision, recall, F1-score, accuracy, goodness-of-fit, and area under the curve.

## Introduction

Even with automation, better working conditions, strict and thorough safety laws, mining is one of the most hazardous occupations in the world^1,2^, and the Indian mining industry is no exception. Overall risk in the Indian mining industry is still beyond the desired levels^3–8^. In general, multiple factors contribute to accidents in the workplace and are typically categorized into two broad categories: unsafe conditions and unsafe behaviors^9–12^. Researchers have proven that unsafe acts are caused by two factors: (1) internal factors such as risk tolerance, risk perception, and self-efficacy^13,14^, and (2) external factors like safety culture, work environment and conditions^15,16^. Most of the existing studies^13,17–25^ are limited to only risk perception in non-mining fields but did not consider the risk tolerance. However, risk tolerance substantially influences decision-making at the workplace when dealing with hazardous conditions. With these motivations, we conducted a comprehensive study on risk tolerance in the Indian coal mine industry context.

Risk tolerance is defined as an individual’s capacity or willingness to accept a certain amount of risk to pursue some goal^26,27^. The term ’risk tolerance’ was first conceived for financial risk decision-making and was defined as the level of risk an individual is willing to take to achieve the desired outcomes^28^. Individuals’ risk tolerance depend on their beliefs, values, and personal goals, which overlap with their feelings of confidence^29^. An individual’s risk tolerance is one of the primary reasons leading to unsafe acts at the workplace, causing various accidents.

**Table 1:** Summary of recent related papers.

Author	Year	Research focus
Calisa et al.^17^	2019	Occupational health and safety management systems depend on a country’s economic, social, cultural, political, and technological basis. Thus, each nation should create its own management system based on its dynamics
Hossam et al.^18^	2016	This research presented a very effective technique for spam filtering by combining the particle swarm optimization and random forest algorithms
Mohamad et al.^19^	2015	This study investigated the application of a hybrid approach by combining the particle swarm optimization and artificial neural network algorithms for predicting the rock unconfined compressive strength
Bi et al.^20^	2022	Safety and reliability analysis of the solid propellant casting molding process based on fuzzy fault tree analysis, particle swarm optimization and back propagation neural network algorithms
Dong et al.^21^	2019	This research studied the sensor network security defense strategy based on attack graph and improved binary particle swarm optimization
Yang et al.^22^	2019	This study presented an intelligent prediction strategy for blasting-induced ground vibration using adaptive neuro-fuzzy inference system optimized by genetic algorithm and particle swarm optimization
Gong et al.^23^	2022	The authors proposed a multi-period portfolio selection method under the coherent fuzzy environment with dynamic risk-tolerance and expected-return levels
Wang et al.^24^	2016	The research focused on identifying the critical factors and paths that influence workers’ safety risk tolerance and to explore how they contribute to accident causal model from a system thinking perceptive
Bhandari et al.^13^	2022	This study leveraged survey data from 11811 construction workers from 19 countries to empirically validate the associations between safety climate, risk tolerance, and risk-taking decisions in the workplace using linear-mixed effects model analysis
Vinnem J.^25^	2021	This work delivered a summarized case study for a fictitious normally unmanned facility, and presented risk results for three different cases with varying extents of safety systems
Reddy et al.^30^	2020	This paper suggests ML algorithms incorporating PCA perform better with high-dimensional datasets. However, ML techniques without dimensionality reduction perform better with low-dimensional datasets
Pei et al.^31^	2022	This work proposes scene graph semantic inference for cross-modal image and text matching. Using visual and textual scene graphs and graph convolutional networks, the technique analyses the local semantic correlations of inter-modal object relationships
Jiang et al.^32^	2021	This paper proposes visual dialogue for establishing semantic relationships between visual and written content. Aligning visual and textual knowledge reduce the gap between modalities. Graph structure connects textual-semantic visual objects

Many studies^14,26,33–35^ have confirmed that an individual with a higher tolerance to risk takes more risks than a less risk-tolerant individual. Lehmann et al.^33^ have demonstrated that the risk tolerance levels in the mining industry significantly influence the risky behavior of male miners. Hunter et al.^26^ have shown that a pilot’s decision-making is substantially influenced by their risk tolerance levels in the aviation industry. Similar conclusions have also been drawn by Bhandari et al.^34^ for the construction sector. Besides, safety professionals associated with inherently hazardous sectors like mining, aviation, construction, chemical, nuclear power, etc., consider risk tolerance as an important factor at the workplace as employees frequently engage in various hazardous activities^14,35^. In particular, they are more concerned about an individual’s risk tolerance level as the consequences of risky decisions can result in catastrophic accidents. Therefore, an objective assessment of risk tolerance among individuals is crucial to persuade them to engage in safer workplace behavior. Hence, it is essential to evaluate and minimize miners’ risk tolerance to improve an organization’s safety framework and overall performance.

To evaluate and reduce risk tolerance, we need to understand why two different individuals under similar workplace conditions and environments behave differently. In particular, we have to comprehensively identify the factors influencing miners’ risk tolerance and select the most critical factors among all the identified factors for objectively assessing miners’ risk tolerance levels. Since the number of factors influencing an individual’s risk tolerance level is large and complex, determining their combined effect on risk tolerance is challenging and time-consuming, especially using conventional methods.

On the other hand, machine learning^7,36^ and soft computing^23^ paradigms can efficiently predict outcomes when complex multi-factor situations are involved. In addition, these paradigms are very helpful in analyzing multivariate data sets in reasonably less time than the traditional statistical methods. Many machine learning and soft computing methods have been widely used for objective assessments and predictions by identifying and analyzing patterns in data. These paradigms have also been used to solve various complex engineering problems and other problems in almost every field of science and life. For example, Guo et al.^37^ used an artificial neural network (ANN) to forecast the capital cost of open cast mining projects, Yang et al.^22^ predicted the ground vibration levels using an adaptive neuro-fuzzy inference system (ANFIS), genetic algorithm (GA) and particle swarm optimization (PSO). Likewise, various other studies, such as Koopialipoor et al.^38^, Jothi et al.^39^, and Zhou et al.^40^ have also utilized different machine learning and soft computing techniques for the prediction purpose. We summarize some recent related papers in Table 1.

**Figure 1:**
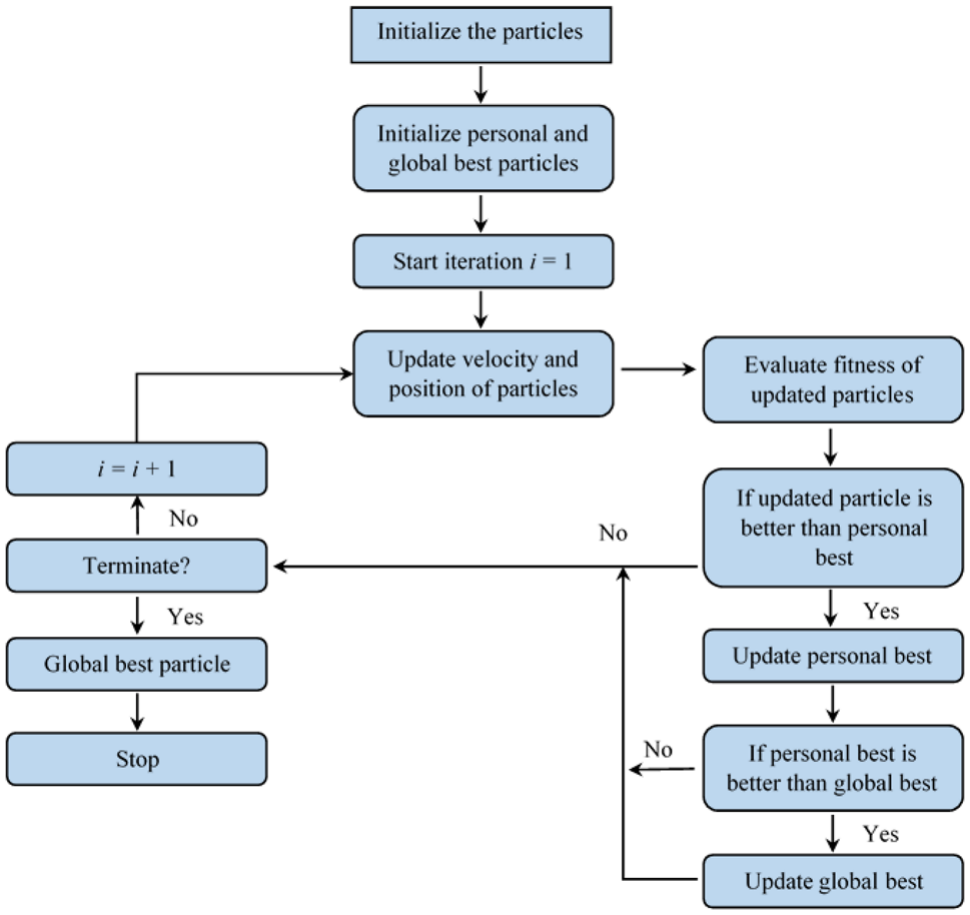
Flowchart of particle swarm optimization (PSO).

In this paper, we aim to predict the risk-tolerance level of miners in the Indian mining industry using a multi-class classifier. In this context, we first identify the exhaustive set of factors (or features) affecting an individual’s risk tolerance. Based on the identified factors, we then conducted one-to-one interviews with 360 employees from three categories, i.e., non-executive, supervisor, and executive of Bharat Coking Coal Limited (BCCL), a major coal-producing company in India, for primary data collection. Next, we propose a wrapper based hybrid system in which we combine particle swarm optimization (PSO)^41^ and random forest (RF)^42^ classifier for training a multi-class classification model using a subset of features. Herein, our objective is to maximize the F1-score of the trained model. In this view, we first divide the primary data into two sets: the training set and the testing set. We then feed the training set into the proposed hybrid PSO-RF system to select a subset of features and fine-tune the classification model using *k*-fold cross validation. We then use the testing set to compare the experimental results of the PSO-RF with support vector machine (SVM)^43^, k-nearest neighbor (kNN)^44^, decision tree (DT)^45^, and RF^42^ algorithms in terms of precision, recall, F1-score, accuracy, goodness-of-fit^46^, and area under the curve (AUC).

The final classification model could be used for categorizing new/existing miners into different groups based on their risk profiling and to guide mine management in order to deploy them in suitable workplace environments. Also, it can be used by the organizations to form safety policies and to design safety training modules based on miners’ risk profiles to reduce risk tolerance and improve the overall safety of an organization.

The rest of the paper is arranged as follows. Section “Preliminaries” provides an overview of PSO, RF classifier, and k-fold cross validation. Section “Identification of factors influencing risk tolerance and data collection” first identifies the exhaustive set of factors influencing miners’ risk tolerance and then describes the dataset along with how we have collected the primary data. The proposed hybrid PSO-RF system and the experimental results are presented in Sections “Proposed hybrid PSO-RF system” and “Experimental results”, respectively. Finally, we conclude the paper in Section “Conclusions”.

## Preliminaries

In this section, we provide overviews of PSO, RF classifier, and $k-$fold cross validation as the proposed hybrid system is based on them.

### An overview of particle swarm optimization (PSO)

Particle swarm optimization (PSO) is a nature-inspired evolutionary optimization technique introduced by Kennedy et al.^41^. PSO solves a given optimization problem by having a set of candidate solutions, known as particles, and iteratively moving particles around the search space based on their position and velocity and a fitness function^47^.

In PSO usually all the particles have same dimension and can produce a complete solution to a given optimization problem. Let the position and velocity of a particle in dimension *d* be denoted by $x_{i,d}$ and $v_{i,d}$, respectively, then the position and velocity vectors of a particle at iteration *t* are denoted as:

1 $$\begin{aligned}{} & {} x_i(t) = [x_{i,1},x_{i,2},\ldots ,x_{i,d}] \end{aligned}$$

2 $$\begin{aligned}{} & {} v_i(t) = [v_{i,1},v_{i,2},\ldots ,v_{i,d}] \end{aligned}$$

In general, the solution obtained by the particles of the swarm is given by their position vectors and the velocity vectors help in updating the position vectors of the particles. The movement of each particle is influenced by both its best-known position and the best-known position among the particles of the swarm, which is expected to drive the particles towards an optimal solution. In each iteration, the velocity and position vectors of each particle are updated using Eqs. 3 and 4, respectively.

3 $$\begin{aligned} v_i(t+1) = w\times v_i(t) + c_1\times r_1 \times (x^p_i - x_i(t)) + c_2\times r_2 \times (x^g_i - x_i(t)) \end{aligned}$$

4 $$\begin{aligned} x_i(t+1) = x_i(t) + v_i(t+1) \end{aligned}$$

where $v_i(t+1)$ and $x_i(t+1)$ are the velocity and position of a particle at iteration $(t+1)$, respectively. $x^p_i$ and $x^g_i$ are the personal and global best solutions, respectively. $c_1$ and $c_2$ are constant acceleration coefficients. $r_1$ and $r_2$ are random variables in range [0, 1]. *w* is the inertial weight. After the last iteration, the PSO returns the global best particle, i.e., the best solution obtained with reference to the given optimization problem and its fitness function. The flow chart of the PSO is depicted in Fig. 1.

### An overview of random forest (RF) classifier

**Figure 2:**
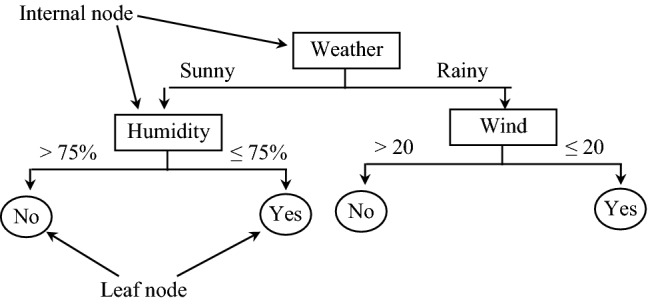
Pictorial illustration of decision tree.

Random forest (RF) or random decision forest classifier^42^ is a popular machine learning algorithm based on supervised learning. To better understand the RF model, we first need to understand the decision tree (DT)^45^, which is the building block of an RF model. In particular, a DT is a flowchart-like arrangement in which the classification rules are defined from root to leaf nodes. Typically, each internal node of the tree depicts attributes (e.g., sunny or rainy weather), each branch depicts all possible values of the attributes, and each leaf node describes the class label. Let us illustrate with this an example. Let us assume that we want to play football on a given day, then we can decide whether to play or not based on the DT as shown in Fig. 2.

Herein, if the given day is sunny and has humidity greater than 75%, then the above shown DT will classify not to play football. Similarly, if the humidity on a sunny day is less than or equal to 75%, then it will be classified as yes, i.e., can play football.

**Figure 3:**
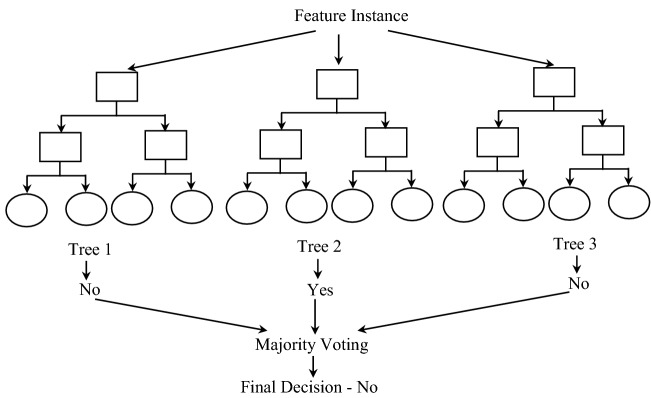
Pictorial illustration of random forest.

The RF creates an ensemble of many individual DTs at training time; each DT in the ensemble predicts a class label, and the class label predicted by the most trees becomes the output of the prediction model. A sample RF classifier with three DTs is shown in Fig. 3 for deciding whether to play football or not on a given day. Herein, the first and third trees predict “No” as the class label, whereas the second tree predicts “Yes” as the class label. Since majority (2 of 3) of trees classify “No” as the class label, the final class label is also “No”, i.e., not to play. Note that RF mitigates the over fitting problem of the DTs by combining prediction of a large number of DTs. Hence, RF generally outperforms the DTs.

### $k-$Fold cross validation

**Figure 4:**
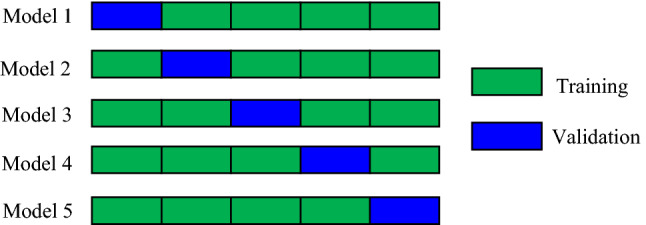
An example of 5-fold cross validation.

Cross validation^48,49^ is a process of resampling the data sets employed to assess the machine learning models. There are three cross validation techniques: random sub-sampling, leave-one-out validation, and k-fold cross validation. In this paper, we adapt $k-$fold cross validation as it generally uses all the observations for both training and validation and has a lower bias than other procedures. In $k-$fold cross validation, we randomly split the training data into *k* approximately equal-sized groups (or folds) and repeat the following operations *k* times. Each time use the $(k-1)$ folds to train the machine learning model and the one fold to validate the trained model. Finally, we average the results of these k machine learning models to produce a single estimation. An example of 5-fold cross validation is as shown in Fig. 4.

## Identification of factors influencing risk tolerance and data collection

Many factors influence an individual’s risk tolerance capacity at the workplace. Some factors have a higher influence on an individual’s risk tolerance capacity than others, whereas others have little to no impact. There are two classes of factors; some increase an individual’s risk tolerance capacity, while others decrease it depending on the situation and the individuals.

According to the Ongoing Professional Practice Evaluation (OPPE)^50^, risk tolerance may depend on a large number of factors. Yin et al.^51^. showed that demographic variables like age, working experience, accident exposure have correlations with coal miners’ safety attitudes. Paul et al.^52^ established that risk-taking behavior is prominent among miners, and production pressure, illiteracy, unawareness of consequences of risky behavior, lack of skills, and trying to save time and efforts make the workers take more risk. Mirzaei et al.^53^ indicated that personal and environmental factors have a higher influence on unsafe practices by miners using the Bayesian network. Similarly, Wang et al.^24^ demonstrated that a worker’s risk tolerance could also be affected by working experiences and knowledge, work characteristics, personal subjective perception, and safety management. Khosravi et al.^54^ concluded that individual attributes like site condition, society, organization, supervision, contractor, project management, and work group factors are some of the causes of unsafe behavior at a construction site.

Manjula et al.^1^ claimed that personal and organizational factors influence the safety behavior of construction workers. In contrast, Man et al.^55^ asserted that behavioral and environmental factors also affect risk-taking behavior apart from personal factors. Tchiehe et al.^56^ identified economic, personal, cultural, political, social, ethical, psychological, and characteristics of the risk as the primary parameters contributing to individuals’ risk tolerance. Inouye^57^ categorized the factors affecting risk tolerance into three levels, i.e., structural or institutional level, community level and psychological level. The author also discussed seven factors that increase the risk tolerance capacity of an employee in an organization and three factors that decrease the tendency to take the risk.

**Table 2:** Brief description of all identified 36 factors.

ID	Factors	Brief description
Organizational factors		
$F_1$	Management commitment	If management is committed to safety, then risk tolerance among employees of such organization is comparatively reduced
$F_2$	Structure and responsibility within the organization	Poor organizational structure, lack of defined roles and reporting mechanism creates a sense of carelessness among employee which ultimately increases their risk tolerance level
$F_3$	Communication and information management within the organization	Two-way communications within the organization, effective feedback mechanism and minimal communication barrier results in increased safety behavior at the workplace thereby decreasing the risk tolerance level of individuals
$F_4$	Safety culture	A positive safety culture within an organization and top priority on overall safety of employees results in a general reduction of risk tolerance levels among employees, leading to improved organizational safety parameters
$F_5$	Supervision	Effective supervision is an important tool for reducing workplace safety violations and accidents which eventually reduces risk tolerance level among employees
$F_6$	Safety regulations and SOPs	Clear and detailed safety regulations and SOPs based on an accurate assessment of risks associated with mining activities helps individual to identify the hazards associated with it and outlines the risk control processes while decreasing the risk tolerance level of employees
$F_7$	Training and competency	Lack of proper training, absence of regular and unbiased assessment of competency results in less than adequate hazard perception among workers leading to higher risk tolerance level and increased involvement in workplace accidents
$F_8$	Welfare	Mining companies providing comprehensive welfare amenities have a better safety record and workers tend to take less risks at the workplace because of better risk perception which eventually reduces the risk tolerance level of individuals
$F_9$	Contract management	Lack of comprehensive contract management, absence of clarity on safety obligations of contractors and the least cost criteria for awarding contracts results in higher risk tolerance level among operators, supervisors and managers
$F_{10}$	Outcome of noncompliance	Violations of safety norms are common and repetitive in the mining industry as the provisions of penalty in safety regulations are outdated and obsolete
$F_{11}$	Allocation of resource	Inadequate allocation of resources in an organization can eventually increase the risk tolerance level among employees and thereby cause unsafe behavior at workplace
$F_{12}$	Acceptance of wrong practices	Repeating a particular type of unsafe practice at the workplace over a long period of time leads to acceptance of such practices as a usual, safe and normal process. Sometime it becomes the most accepted way of performing any particular activity, which ultimately increases the level of risk tolerance level among employees
$F_{13}$	Acceptance of LTA safety in design of equipment and processes	Accepting less than adequate safety parameters in designing equipment and processes leads to high risk tolerance levels, which in turn creates unsafe conditions at the workplace
$F_{14}$	Availability of PPE and other safety equipment	Non-availability of sufficient and suitable Personal Protective Equipment (PPE) compels workers to take more risks than necessary and over a period of time they start ignoring the importance of PPE due to an increase in their risk tolerance level
$F_{15}$	Culture of denial	Culture of denial is about beliefs or misconception that everything is fine and nothing will happen. Due to a culture of denial that exists in organizations, the management tends to ignore the abnormalities reported to it which increases the exposure of workers to higher risk activities thereby increasing the risk tolerance of an organization as a whole
$F_{16}$	Decision motivation	Motivational measure like safety incentives as cash rewards enhances positive attitude of workers towards safety which eventually lowers the risk tolerance level among employees
Human factors		
$F_{17}$	Age	In general, knowledge, experience and job skill of a worker varies in proportion to their age, whereas physical strength, reflex action or response etc. varies inversely with age of individuals
$F_{18}$	Gender	It is evident that significant biological differences exist between male and female workers which results in variation in their attitudes and behavior towards risk inherent activities
$F_{19}$	Physical health and condition	Health problems like high blood pressure, lack of sleep and other health problems or diseases severely affect an individual’s risk tolerance which affects working quality and safety performance of individuals
$F_{20}$	Educational background	Educated employees of any organization possess a positive outlook towards safety and health programs. Hence, it has been observed that educated employees have low risk tolerance levels
$F_{21}$	Marital status	Family responsibility of married workers motivate them to take less risks at the workplace and hence married workers have lower risk tolerance levels than unmarried workers with comparatively less responsibility
$F_{22}$	Professional knowledge and working experience	Knowledgeable professionals act more rationally and have a lower tolerance for risk at workplace
$F_{23}$	Alcoholism	Alcoholism or drinking habits not only have an adverse effect on the health and well-being of workers, but also cause deterioration in their overall performance. Alcohol influences an individual’s moods, emotions, actions, reactions and decision-making ability which impacts their risk tolerance capacity
$F_{23}$	Previous exposure to accidents	Employees with previous exposure to accident or near miss incidents or any other similar incidents have lower risk tolerance levels
$F_{25}$	Overconfidence	Over confident individuals may take higher risk without proper assessment of a risky situation or identification of hazards and consequently make irrational decisions
$F_{26}$	Job satisfaction	If employees in an organization are satisfied with their jobs, then it will make them highly focused on their tasks. Such employees make fewer mistakes at the workplace
$F_{27}$	Personality	Personality traits primarily influence an individual’s attitude, behavior and decisions. Individuals with different personality traits have different attitude and behavior regarding their safety. Some personality traits positively influence risk tolerance levels while others influence it negatively
$F_{28}$	Risk perception	Individuals with higher risk perception are likely to become more conscious about risks and subsequently develop lower risk tolerance levels. Risk tolerance is inversely proportional to risk perception
$F_{29}$	Judgement ability	Judgment ability is the process by which individuals evaluate evidence to assess the likelihood of different outcomes. At a workplace, judgment ability helps workers to analyze and comprehends problems or situations according to their knowledge and experience. Judgment ability acts as an important parameter of risk tolerance
$F_{30}$	Familiarity with a task	Familiarity with a task grows with successful execution of a particular task several times. However, such unconscious competencies sometimes lead to growing complacency resulting in blind spots to perceive potential hazards, which eventually increases the risk tolerance level of individuals or the organization as a whole
$F_{31}$	Over-trust on the equipment	Over-trust on any equipment or system grows over a period of time if frequency of failure is less. However, such over-trust sometimes influences the risk tolerance level of the operator or supervisor
Task condition and environment factors		
$F_{32}$	Site layout and housekeeping	Workers of mines where the working sites are well planned and designed after considering the health, and safety of workers, become less risk tolerant over a period of time, as they become used to working in a safe working environment
$F_{33}$	Workload and time constraints	Higher production target or too much work load may pressurize employee to ignore and circumvent safety standards and guidelines at the workplace to achieve set targets
$F_{34}$	Lack of adequate safety provision	Due to defective design or lack of ergonomic design, the workers, particularly operators of heavy equipment include wrong practices in their processes of operating heavy machinery. Adopting such inefficient practices without any negative outcome creates a false sense of security which eventually increase their risk tolerance levels
Social factors		
$F_{35}$	Peer pressure	A person’s decisions and actions are significantly influenced by the people around them. Peer pressure can affect an individual in both positive or negative direction with respect to their safety
$F_{36}$	Socioeconomic condition	It is a well-established fact that overall attitudes of the society influence an individual towards every aspect of life

Based on the above-discussed findings, we identify and compile a total of thirty-six factors that influence an individual’s risk tolerance with respect to the mining industry and divide them into four major groups: (1) organizational factors, (2) human factors, (3) task condition and task environment factors, and (4) social factors.

We provide brief descriptions of each identified factor in Table 2. Based on these factors, we next generate a questionnaire with forty two (42) questions to perform a survey among miners (or respondents) for data collection. The questions require respondents to answer them based on discrete categories or levels. In general, we divide the questionnaire into three parts. The first part includes three (4) questions to collect the personal information of respondents, i.e., designation, age, gender, and work experience. The second part contains one (1) question to know the type of risks miners usually take and one question to categorize the miners into five classes of risk tolerance:

- **Class 1:** Very less risk-tolerant.
- **Class 2:** Less risk-tolerant.
- **Class 3:** Moderate risk-tolerant.
- **Class 4:** High risk-tolerant.
- **Class 5:** Very high risk-tolerant.

Herein, the very less risk-tolerant class represents respondents who take the risk at the workplace once a year, the less risk-tolerant class signifies individuals who take a risk once a month, moderate risk-tolerant class means miners who take risk twice a week, high risk-tolerant class denotes respondents who take the risk every other day, and very high risk-tolerant class indicates individuals who take risk daily.

The third part includes thirty-six (36) closed-ended questions to capture how much each factor (or feature) influences an individual to take the risk on a Likert scale ranging from one to five, with one meaning minimum influence and five denoting maximum influence.

In this paper, we utilized the stratified random sampling method to conduct the survey and reduce skewness and biasness in the collected data. The stratified random sampling method divides the members of a population into smaller sub-groups known as strata before sampling such that each individual of the population is allotted to only a single stratum. In particular, we conducted the survey among 360 employees of BCCL from three classes: non-executive, supervisor and executive, using simple random sampling. The non-executive class comprises non-executive employees other than the supervisory staff, and the supervisor class consists of mining sardars, overmen, and foremen. In contrast to these, the executive class includes all the officers and mine managers. Among the selected 360 respondents, 352 were males (i.e., $97.77\%$), and only 8 were females (i.e., $2.23\%$). The mean age of selected miners was 36 years, and the mean duration of their work experience was 14 years.

This paper considers the responses to the third and second parts of the questionnaire as the complete feature set and the class labels, respectively, to model the risk-tolerance prediction problem as a multi-class classification problem. In other words, the data set has 606 instances, each with 36 features and a class label among five classes of risk tolerance discussed above. The complete set of features and class labels are denoted as $F = \{F_1,F_2,\ldots , F_{36}\}$ and $C = \{C_1,C_2,C_3,C_4,C_5\}$, respectively.

## Proposed hybrid PSO-RF system

**Figure 5:**
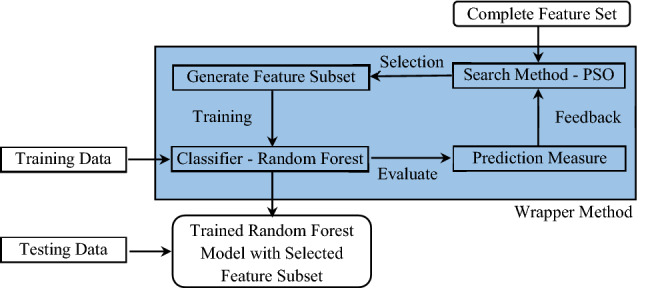
Proposed wrapper based hybrid system based on PSO and RF classifier.

As mentioned earlier, we intend to predict the risk-tolerance level of coal miners in the Indian mining industry. In this paper, we train a multi-class classifier using the wrapper based feature selection^58^ method to reduce the dimension of the dataset and optimize the trained model for maximizing the F1-score. There exist two basic requirements for a wrapper based feature selection strategy, i.e., a search algorithm and an objective function. In particular, wrapper methods select the best feature subset by iteratively generating different combinations of the features using the search algorithm and training a specific machine learning model to evaluate the usefulness of the generated feature subsets with respect to an objective function, as shown in Fig. 5.

This paper utilizes the particle swarm optimization (PSO) as the search algorithm, random forest (RF) as the classifier, F1-score as the objective function, and propose a hybrid PSO-RF system to classify the miners. To this end, we first divide the complete dataset into two parts: training data and testing data. Next, we feed the training set to the proposed hybrid PSO-RF system which runs for a given number of iterations. In each iteration, the proposed system generates several feature subsets using the PSO, trains an RF classifier for each subset using $k-$fold cross validation to avoid over fitting, evaluates the RF model based on the F1-score, and stores the model yielding the maximum F1-score so far. Once the specified number of iterations are elapsed, it outputs the final optimized RF classifier with the selected subset of features. Finally, we evaluate the obtained RF model using the testing data and various performance metrics. Now, we present the particle representation scheme, the derivation of the objective function, i.e., F1-score, and a case study to illustrate the overall working of the proposed system.

### Particle representation scheme

In this section, we explain the particle encoding and decoding schemes to generate various feature subsets in proposed hybrid PSO-RF system. In particular, we encode a particle as a $2-$dimensional array in which each column corresponds to a feature, for example, the first column stands for first feature ($F_1$), the second column stands for second feature ($F_2$) and so on. We initialize each element of a particle with a random number $p \mid -5 \le p \le 5$, following the uniform distribution.

Let us consider an illustrative example to understand the encoding and decoding scheme in detail. Suppose there exist 13 features in the complete dataset, then a particle is encoded as follows.

**Table:** 

$F_1$	$F_2$	$F_3$	$F_4$	$F_5$	$F_6$	$F_7$	$F_8$	$F_9$	$F_{10}$	$F_{11}$	$F_{12}$	$F_{13}$
2.2	1.0	− 3.0	− 5.0	− 2.3	2.9	− 1.5	− 2.9	− 1.6	− 3.6	1.0	1.4	3.3

Next, let us understand how we decode this particle to know which features to retain and discard, respectively. To decode the particle, we check value corresponding to each feature, and if it is less than or equal to zero, then we convert it into a binary zero, i.e., we discard the feature. On the other hand, if the particle value of a feature is greater than zero, then we interpret it as a binary one and retain the feature. The decoded particle is shown below.

**Table:** 

$F_1$	$F_2$	$F_3$	$F_4$	$F_5$	$F_6$	$F_7$	$F_8$	$F_9$	$F_{10}$	$F_{11}$	$F_{12}$	$F_{13}$
1	1	0	0	0	1	0	0	0	0	1	1	1

Notice that the values of features $F_1$, $F_2$, $F_6$, $F_{11}$, $F_{12}$, and $F_{13}$ are transformed into binary one and the values of features $F_3$, $F_4$, $F_5$, $F_7$, $F_8$, $F_9$, and $F_{10}$ are inferred as binary zero. This implies that the feature subset $F_r = \{F_1, F_2, F_6, F_{11}, F_{12}, F_{13}\}$ is retained while the feature subset $F_d = \{F_3, F_4, F_5, F_7, F_8, F_9, F_{10}\}$ is discarded.

### Derivation of objective function

In this section, we define the objective function that we use to evaluate the worth of the feature subsets. To derive the objective function, we first depict the confusion metric^58^ for two class (or binary) classification problem as Table 3.

**Table 3:** Confusion matrix for binary classification with respect to Class 1, where “Class 1 (C$_1$)” and “Class 2 (C$_2$)”.

	Predicted class		
Actual class		C$_1$	C$_2$
	C$_1$	TP	FN
	C$_2$	FP	TN

Let us first describe the meanings of true positive (TP), true negative (TN), false positive (FP), and false negative (FN).

- **TP:** it denotes to the number of predictions where class 1 is classified as class 1.
- **TN:** it denotes the number of predictions where class 2 is classified as class 2.
- **FP:** it denotes the number of predictions where class 2 is classified as class 1.
- **FN:** it denotes number of predictions where class 1 is classified as class 2.

We now briefly describe the precision, recall, accuracy, and F1-score using above mentioned confusion matrix as follows.

#### Accuracy

It provides the overall accuracy of the classification model and is defined as the fraction of the total number of predictions that were correct.

5 $$\begin{aligned} Accuracy = \frac{TP+TN}{TP+TN+FP+FN} \end{aligned}$$

**Figure 6:**
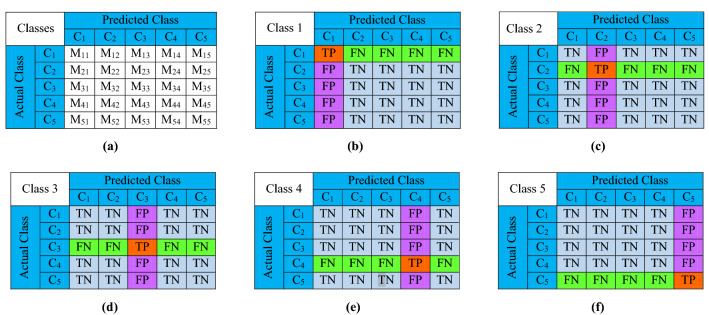
Confusion matrix for (**a**) Generic Interpretation, (**b**) Class 1, (**c**) Class 2, (**d**) Class 3, (**e**) Class 4, and (**f**) Class 5.

#### Precision

It quantifies the fraction of predictions as class 1 were actually class 1. In other words,

6 $$\begin{aligned} Precision = \frac{TP}{TP+FP} \end{aligned}$$

#### Recall

It computes the fraction of predictions of all class 1 samples were correctly predicted as class 1, i.e.,

7 $$\begin{aligned} Recall = \frac{TP}{TP+FN} \end{aligned}$$

#### F1-score

It combines the precision and recall by taking their harmonic mean. In other words,

8 $$\begin{aligned}{} & {} F1{\text{-}}score = \frac{2 \times Precision \times Recall}{Precision + Recall} \end{aligned}$$

9 $$\begin{aligned}{} & {} F1\text {-score} = \frac{2 \times TP }{2 \times TP + FP + FN} \end{aligned}$$

Usually, a classification model with higher accuracy, precision, recall, and F1-score is considered better than one with lower accuracy, precision, recall, and F1-score.

However, in this paper, we deal with a multi-class classification problem for which the confusion matrix^59^ interpretation is shown in Fig. 6. Herein, each row represents an actual class, i.e., the first row depicts $C_1$, the second row denotes $C_2$, and so third. Likewise, each column represents a predicted class, i.e., the first column denotes $C_1$, the second column depicts $C_2$, and so third. Now, we explain the process of calculating the *TP*,*FN*, *FP*, and *TN* as follows.

In general, for a given class $C_k$, the number of $TP_k$ is the value of the cell on the $k^{th}$ row and column, i.e.,

10 $$\begin{aligned} TP_k = M_{kk} \end{aligned}$$

The number of $FN_k$ is the sum of the values of all the cells on $k^{th}$ row, except the value of the cell on $k^{th}$ column, i.e.,

11 $$\begin{aligned} FN_k = \sum _{i=1 \wedge i \ne k}^{|C|} M_{ki} \end{aligned}$$

The number of $FP_k$ is sum of the values of all the cells on $k^{th}$ column, excluding the value of the cell on $k^{th}$ row, i.e.,

12 $$\begin{aligned} FP_k = \sum _{i=1 \wedge i \ne k}^{|C|} M_{ki} \end{aligned}$$

The number of TN$_k$ is the sum of the values of all the cells, except the values of the cells on $k^{th}$ row and column, i.e.,

13 $$\begin{aligned} TN_k = \sum _{i=1 \wedge i \ne k}^{|C|} \sum _{j = 1 \wedge j \ne k}^{|C|} M_{ij} \end{aligned}$$

In this paper, we want to examine the trained classification model with respect to all the classes using a single parameter. Hence, we consider the micro F1-score as the objective function, i.e.,

14 $$\begin{aligned} \text {Micro}~F1\text {-score} = \frac{2 \times \sum _{k=1}^{|C|}TP_k}{2 \times \sum _{k=1}^{|C|} TP_k + \sum _{k=1}^{|C|} FP_k + \sum _{k=1}^{|C|} FN_k} \end{aligned}$$

This may be noted that the Micro F1-score will be referred as F1-score in the rest of the paper.

## Experimental results

**Figure 7:**
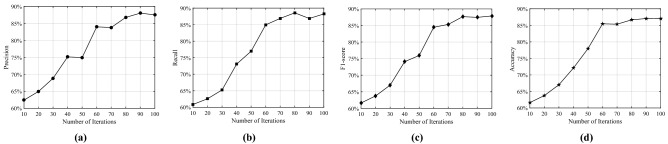
Convergence curves of PSO-RF algorithm for (**a**) Precision, (**b**) Recall, (**c**) F1-score, and (**d**) Accuracy.

We implemented both pure RF and the proposed hybrid PSO-RF on a system with a Windows 10 Standard 64 bits operating system with Intel(R) Core TM i7-8550U CPU @1.80 GHz 2.00GHz and 8.00 GB of RAM using Python 3.8. As mentioned before, the raw dataset has 360 instances with 36 features and 5 classes, which was divided into two subsets: training set (270 instances) and testing set (90 instances). We use the training set for selecting features and training the classifiers, whereas the testing set is used for the final evaluation of the trained models. During the feature selection phase, we considered 100 particles, 200 iterations, 100 trees, and 5-fold cross-validation for the proposed PSO-RF algorithm. In PSO, we kept the same parameters as used in^41^. The convergence curves of the PSO-RF algorithm for precision, recall, F1-score, and accuracy are shown in Fig. 7. Herein, the values of precision, recall, F1-score, and accuracy increase till 60 iterations. After that, there is a slight variation in their values, signifying that the PSO-RF converges.

**Figure 8:**
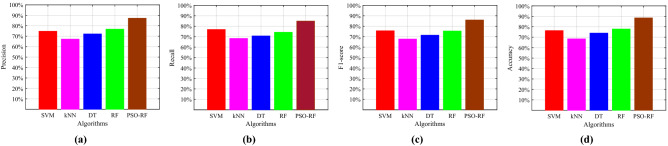
Comparison of different algorithms for (**a**) Precision, (**b**) Recall, (**c**) F1-score, and (**d**) Accuracy.

After that, we compare the experimental results of PSO-RF algorithm with four traditional classification algorithms, namely SVM with RBF kernel, kNN wih $k=5$, DT with J48 implantation, and RF model. To begin with, we depict the precision of all the algorithms in Fig. 8a. It is easy to observe that the PSO-RF attains superior performance as compared to other four algorithms, i.e., it attains maximum precision. In particular, it has $12.44\%$, $19.96\%$, $15.01\%$, and $10.42\%$ more precision than that of SVM, kNN, DT, and RF, respectively. Next, we assess all the algorithms in terms of recall as shown in Fig. 8b. Notice that the recall of the PSO-RF is $8.07\%$, $16.63\%$, $14.24\%$, and $10.57\%$ more than that of SVM, kNN, DT, and RF, respectively. In Fig. 8c, we depict the experimental results of all algorithms in terms of the F1-score. The PSO-RF has $10.26\%$, $18.29\%$, $14.64\%$, and $10.57\%$ more F1-score than the SVM, kNN, DT, and RF, respectively. In similar fashion, the accuracy achieved by the all the algorithms is presented in Fig. 8d. In particular, the PSO-RF achieves $12.16\%$, 20.06, $14.57\%$, and $10.67\%$ higher accuracy than SVM, kNN, DT, and RF, respectively.

**Figure 9:**
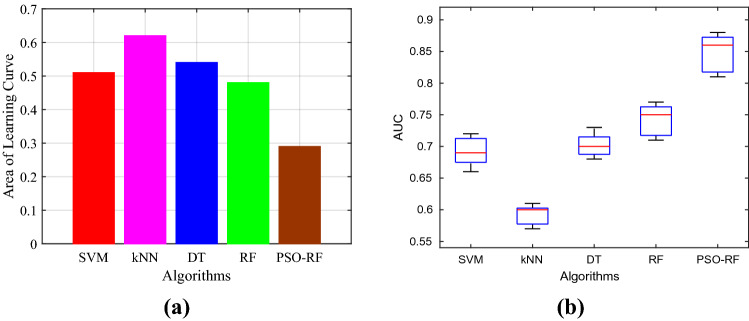
Comparison of different algorithms for (**a**) Area of Learning Curve and (**b**) AUC.

Finally, we compare the algorithms in terms of goodness-of-fit and the AUC. The goodness-of-fit enables us to assess the steepness of the learning curve of a classification algorithm. It is defined as the region between the highest accuracy and learning curve. Note that the lesser the area under goodness-of-fit, the better the algorithm is. Fig. 9a shows the normalized (between 0 and 1) area under goodness-of-fit of the algorithms under consideration. It is clear that the PSO-RF has the minimum area among all the algorithms. In Fig. 9b, we show the AUC of different algorithms using box plot. The more the AUC, the better the classification method is. Notice that the AUC of the PSO-RF is maximum among all the algorithms. This is because the PSO-RF is enabled with a feature selection method, whereas the SVM, kNN, DT, and RF algorithms do not employ any feature selection strategy.

## Conclusions

The coal mining industry has an inherent risk of workplace accidents and hazards. It is the unsafe act which accounts majority of the accidents at workplace. The unsafe act of miners is mainly dependent on their risk perception and risk tolerance level. In this study, we first conducted one-on-one interviews with 360 miners of BCCL and divided them into five classes of risk tolerance. We then presented a wrapper based hybrid PSO-RF system to select a subset of features and train a multi-class classifier with the aim of maximizing the F1-score. We then compared the experimental results of PSO-RF with SVM, kNN, DT, and RF algorithms to assess its efficacy in terms of precision, recall, F1-score, accuracy, area of the learning curve, and AUC. The results revealed that compared to the conventional algorithms, the PSO-RF has up to $19.96\%$, $16.63\%$, $18.29\%$, and $20.06\%$ more precision, recall, F1-score, and accuracy, respectively. In addition, the PSO-RF has the minimum area under the goodness-of-fit and maximum AUC among all the compared algorithms.

The outcome of the study will help organizations or safety professionals engaged in risk intensive industries as follows. The recruitment process of an organization can include a provision to determine the risk tolerance level of each employee using the presented model. Based on outcome of the prediction model, the deployment of employees according to their risk tolerance profiles can be done. If organizations periodically evaluate the risk tolerance profile of its employees, they can utilize this information to design robust safety policies and reporting measures for its employees to follow during operations. Once an individual’s or group’s risk profile have been determined, their training needs and modules can be tailored to inculcate appropriate safety behavior at the workplace, with the objective of reducing risk tolerance levels across the organization.

This may be noted that this study was a pilot scale project in which the data was collected from a limited number of employees belonging to a particular workplace. So, it may not be applicable for other workplaces, if there is significant variance in the nature of work, manpower or work environment. In the future, we will extend this study by including a large number of miners from various coal producing companies in India. We will also explore different machine learning and artificial intelligence techniques to improve and generalize the prediction model.

## Data Availability

The datasets used and analyzed during the current study available from the corresponding author on reasonable request.
